# Disrupted Coordination of Hypoglossal Motor Control in a Mouse Model of Pediatric Dysphagia in DiGeorge/22q11.2 Deletion Syndrome

**DOI:** 10.1523/ENEURO.0520-19.2020

**Published:** 2020-10-09

**Authors:** Xin Wang, Anastas Popratiloff, Zahra Motahari, Anthony-Samuel LaMantia, David Mendelowitz

**Affiliations:** 1 Institute for Neuroscience, The George Washington University, Washington, DC 20037; 2 Department of Pharmacology and Physiology, The George Washington University, Washington, DC 20037; 3 Department of Anatomy and Cell Biology, The George Washington University, Washington, DC 20037; 4 Fralin Biomedical Research Institute at Virginia Tech-Carilion School of Medicine, Roanoke, VA 24016; 5 Department of Biological Sciences, Virginia Tech, Blacksburg, VA 24060

**Keywords:** 22q11.2 deletion/DiGeorge syndrome, brainstem circuitry, hypoglossal motor neuron, pediatric dysphagia, whole-cell recording

## Abstract

We asked whether the physiological and morphologic properties of hypoglossal motor neurons (CNXII MNs) that innervate protruder or retractor tongue muscles are disrupted in neonatal *LgDel* mice that carry a heterozygous deletion parallel to that associated with DiGeorge/22q11.2 deletion syndrome (22q11.2DS). Disrupted coordination of tongue movement in *LgDel* mouse pups may contribute to suckling, feeding, and swallowing (S/F/S) disruptions that parallel pediatric dysphagia in infants and toddlers with 22q11.2DS. Using an *in vitro* rhythmically active medullary slice preparation, we found spontaneous firing as well as IPSC frequency differed significantly in neonatal *LgDel* versus wild-type (WT) protruder and retractor CNXII MNs that were identified by retrograde tracing from their target muscles. In response to respiration-related activity, initiation and decay of transiently increased firing in WT protruder MNs is delayed in *LgDel*, accompanied by altered excitatory/inhibitory (E/I) balance. In addition, *LgDel* retractor MNs have a transient increase in firing with diminished IPSC frequency that is not seen in WT. There were no significant differences in cell body volume of either XII class in WT and *LgDel*. Sholl analysis showed the total numbers of dendritic intersections (at 50- and 90-μm radii from the cell soma) were significantly greater for *LgDel* versus WT retractor MNs. Thus, the physiological, synaptic and cellular properties of distinct classes of CNXII MNs that coordinate tongue movement in neonatal WT mice are altered in *LgDel*. Such changes could contribute to sub-optimal coordination of S/F/S that underlies pediatric dysphagia in 22q11.2DS.

## Significance Statement

Pediatric dysphagia is a frequent and serious clinical complication in up to 85% clinically defined neurodevelopmental disorders, including DiGeorge/22q11.2 deletion syndrome (22q11.2DS). Heterozygous deletion of the full set of mouse orthologues of human 22q11 genes disrupts key physiological properties of distinct classes of hypoglossal motor neurons (CNXII MNs) that coordinate oropharyngeal function in the *LgDel* mouse model of 22q11DS, the only established genetic model for pediatric dysphagia. We found significant differences in firing activity, synaptic inputs, and morphology when comparing protruder and retractor CNXII MNs from *LgDel* and wild-type (WT) animals. Our observations provide a foundation for understanding infant hypoglossal nerve as well as tongue function or dysfunction, and their contribution to perinatal feeding and swallowing abnormalities in 22q11.2DS.

## Introduction

All mammals must breathe, suckle and swallow at birth, and disruption of this essential behavior, pediatric dysphagia, is a serious complication for newborns delivered prematurely or with neurodevelopmental disorders (LaMantia et al., 2016; Maynard et al., 2020). Appropriate tongue movement, involving coordinated activity of hypoglossal motor neurons (CNXII MNs) controlling the antagonistic protruder and retractor tongue muscles is essential for optimal suckling and swallowing (Naganuma et al., 2001; Fregosi and Ludlow, 2014). We asked whether changes in the physiological and circuit properties of these CNXII MN classes accompany suckling, feeding, and swallowing (S/F/S) difficulties in *LgDel* mouse pups, a genomically accurate model (Merscher et al., 2001; Meechan et al., 2015) that recapitulates pediatric dysphagia seen frequently in the common neurodevelopmental disorder DiGeorge/22q11.2 deletion syndrome (22q11DS; Eicher et al., 2000; Karpinski et al., 2014).

Protruder muscles move the tongue forward, retractors compress and pull it backwards (Fregosi and Ludlow, 2014), and although antagonistic in nature, both groups cooperate to shape the tongue and form proper food boluses, and swallowing sequences (Naganuma et al., 2001). Protruder MN activity decreases upper airway resistance and is highest during the inspiration phase of respiration (Fuller et al., 1999; John et al., 2005). Retractor MNs may also fire during inspiration (Powell et al., 2014); however, other data indicate they are recruited only during inspiration accompanied by hypercapnia (sleep apnea) aiming to decrease airway collapsibility, while protruder activity dilates the airway (Dobbins and Feldman, 1995). Retractor and protruder MN activity during respiration has been analyzed exclusively in adults. To our knowledge, there is no information on these CNXII MN classes in neonates, despite their likely contribution to the critical suck/breathe/swallow sequence (Matsuo and Palmer, 2015). The disruption of cranial nerve development in *LgDel* from midgestation onward suggests that perinatal cranial nerve dysfunction, including that of CNXII MNs, may be an important contributor to dysphagia pathology because of 22q11.2 deletion (Vitelli et al., 2002; LaMantia et al., 2016).

To better understand the functional consequences of disrupted cranial nerve and circuit development critical for optimal S/F/S in *LgDel* neonates, we assessed, using an *in vitro* rhythmically active medullary slice preparation, the electrophysiological properties and cellular morphology of CNXII MNs with defined projections that are critical for optimal coordination of tongue movements as well as their integration with the suck/breathe/swallow cycle. We found that CNXII protruder and retractor MNs are significantly different in newborn *LgDel* compared with wild-type (WT) mice. These observations provide a foundation for understanding the role of diminished 22q11.2 gene dosage in potential disruption of tongue movements, their coordination with respiratory cycles and altered CNXII function. Such changes in cranial nerve MN circuits could contribute to perinatal S/F/S difficulties in 22q11DS.

## Materials and Methods

All animal experiments were performed in accordance with NIH and Institutional Animal Care and Use Guidelines, approved by the George Washington University Institutional Animal Care and Use Committee (protocol A324). Heterozygous adult *LgDel* males (C57BL6N background) were bred to C57BL6N WT females. A total of 81 pups (male: WT 24, *LgDel* 18; female: WT 27, *LgDel* 12) from 26 litters were used. Fluorescent tracers were injected into the tongue of postnatal day (P)1 pups (birth, P0 based on presence of a litter during daily cage checks) to retrogradely label protruder and retractor CNXII MNs (Fleury Curado et al., 2018). Electrophysiological experiments on P3–P6 pups were double-blinded to genotype, and groups identified subsequently via PCR genotyping.

### Retrograde labeling of protruder and retractor CNXII MNs

**Figure 1.**
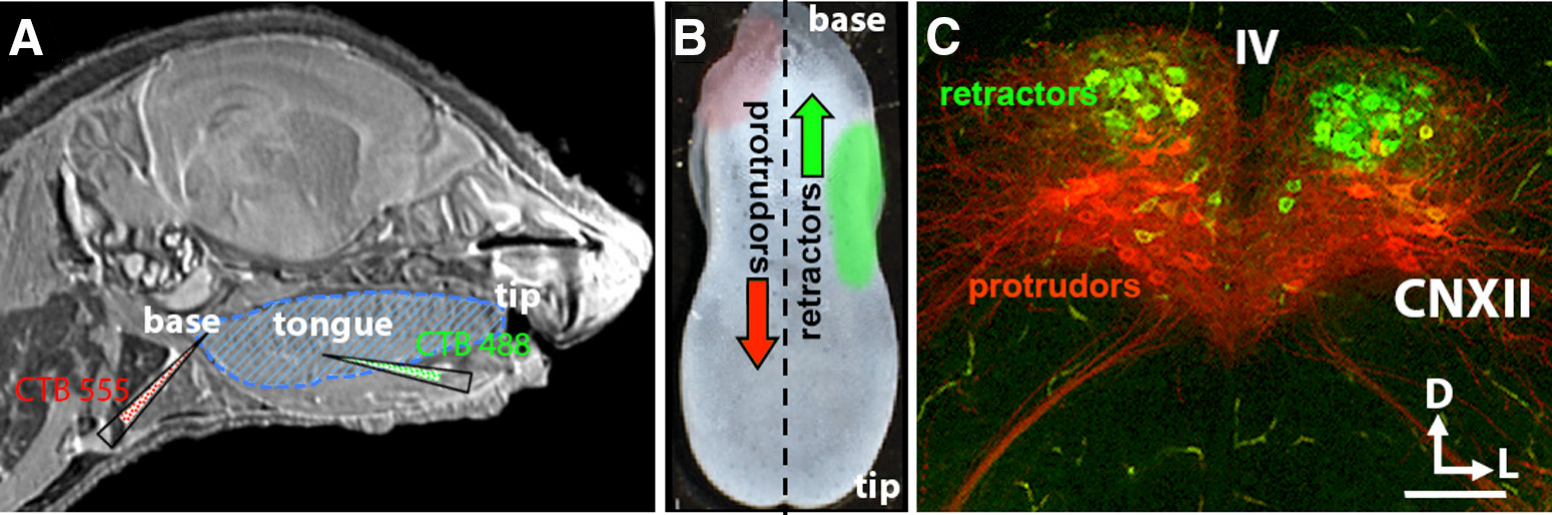
Protruder and retractor hypoglossal MNs in neonatal brainstem slices. ***A***, Sagittal MRI of a neonatal mouse pup (courtesy G. A. Johnson, with permission) showing the tongue (blue hatching) and sites of fluorescent-conjugated cholera toxin injections to retrogradely label protruder (CTB555: red) and retractor (CTB488: green) MNs. ***B***, Neonatal mouse tongue showing the location of protruder and retractor muscles. The base of the tongue is toward the top, the tip toward the bottom of the panel, and the dotted line marks the midline of the dorsal surface. The red shading near the tongue base indicates the general location of protruder muscles, and the green shading on the midlateral aspect indicates the general location of retractor muscles. ***C***, Confocal image of a transverse coronal brainstem section with retrogradely labeled protruder CNXII MNs (red) and retractor (green) CNXII MNs. The ventral CNXII has a higher density of protruder MNs; the dorsal subdivision has a higher density of retractor MNs. Scale bar: 70 μm. D: dorsal, L: lateral.

To identify protruder and retractor CNXII MNs for recording and injection, a two-stage procedure was used. In an initial surgery, P1 pups were anesthetized with the short acting inhalation anesthetic isoflurane, tongues pulled gently from the mouth, and cholera toxin subunit B (CTB) conjugated to Alexa Fluor 555 or 488 (1 μl, 1%, Invitrogen, C22843 and C22841) injected into the base or side of the tongue, respectively (Fig. 1*A*). Protruder MNs were identified by presence of fluorescent tracer (red; CTB-Alexa Fluor 555) and specificity confirmed by absence of labeled brainstem neurons when CTB-Alexa Fluor 555 was injected into the base of the tongue after the medial branch of CNXII was sectioned (*n* = 3). Retractor hypoglossal MNs were identified simultaneously by fluorescent tracer (green; CTB-Alexa Fluor 488) and specificity confirmed by absence of labeled brainstem neurons when CTB-Alexa Fluor 488 was injected into one side of the tongue after the lateral branch of CNXII has been sectioned (*n* = 3).

### Acute rhythmic bursting slice preparation

At P3–P6, pups injected with fluorescent tracers on P0 were anesthetized by isoflurane and killed by rapid cervical dislocation. Brains were submerged in 4°C, continuously 100% O_2_-saturated buffer: 140 mm NaCl, 5 mm KCl, 2 mMCaCl_2_, 5 mm glucose, and 10 mm HEPES. The brains were oriented for vibratome sectioning with caudal ends up and rostral sides attached to an agar block perpendicular to the blade. A single 700-μm slice of the medulla was prepared containing the fundamental respiratory control circuit: hypoglossal motor nucleus and rootlets, intermediate reticular formation interneurons, and pre-Bötzinger complex (Smith et al., 1991). Slices were incubated in NMDG recovery solution: 93 mm NMDG, 93 mm HCl, 2.5 mm KCl, 1.2 mm NaH_2_PO_4_, 25 mm NaHCO_3_, 20 mm HEPES, 25 mm D-glucose, 10 mm MgSO_4_, and 0.5 mm CaCl_2_ saturated by 95% O_2_/5% CO_2_ at 34°C for 10 min, then moved to a recording chamber (10 ml), perfused with room temperature artificial CSF (aCSF):125 mm NaCl, 3 mm KCl, 2 mm CaCl_2_, 26 mm NaHCO_3_, 5 mm glucose, and 5 mm HEPES equilibrated with 95% O_2_ and 5% CO_2_, pH 7.4 (5–10 ml/min; 24–25°C). In a subset of slices, intracellular injections of a highly photostable far-red (excitation laser 640 nm) biocytin fluorophore conjugated dye, CF640R were made (2%; Biotium) to visualize cellular and dendritic morphology.

### Electrophysiology patch clamp techniques

Individual-labeled protruder (red) or retractor (green) CNXII MNs were identified by epifluorescence microscopy, then imaged for recording using differential interference contrast optics (40× water immersion objective, Zeiss Axioskop microscope, Carl Zeiss Inc.). Infrared illumination and high-resolution infrared-sensitive video imaging was used to position the patch pipette on the surface of identified protruder or retractor MNs. To isolate inhibitory or excitatory currents, patch electrodes (thin wall glass capillaries, tip resistance ∼3–4 MΩ; World Precision Instruments) were filled with 150 mm KCl, 4 mm MgCl_2_, 10 mm EGTA, 2 mm Na-ATP, and 10 mm HEPES or 150 mm K-gluconic acid, 10 mm HEPES, 10 mm EGTA, 1 mm MgCl_2_, and 1 mm CaCl_2_ at pH 7.3. A biocytin-conjugated fluorophore, CF640R (2%), was included in the patch solution to label neurons for confocal imaging. Spontaneous and inspiratory related AP activity was analyzed in current clamp; spontaneous and inspiratory related synaptic events were recorded in voltage clamp at −80-mV holding potential. An PV830 Pneumatic PicoPump pressure delivery system (WPI) was used for focal ejection of drugs from a patch pipette positioned within 30 μm from the patched hypoglossal MNs. Prior work has shown this approach effectively delivers drugs 100–120 μm from the pipette tip forward and considerably less behind the drug pipette (Wang et al., 2002). Glutamatergic EPSCs were isolated by focally applying both gabazine (25 μm) to block GABAergic IPSCs and strychnine (1 μm) to block glycinergic IPSCs. GABAergic IPSCs were isolated by focally applying strychnine (1 μm) to block glycinergic IPSCs and D(-)-2-amino-5-phosphopentanoic acid (APV; 20 μm) and 6-cyano-7-nitroquinoxaline-2,3-dione (CNQX; 20 μm) to block glutamatergic excitatory neurotransmission. At the end of each experiment gabazine, or APV and CNQX, were applied to block and confirm the isolation of GABAergic IPSCs and glutamatergic EPSCs, respectively.

### Single-cell injections and imaging

Morphologic properties of protruder and retractor CNXII MNs filled intracellularly with biocytin (CF640R) were analyzed in confocal images stacks obtained at low (10×) and high (20×) magnification. Biocytin fluorophore was confined to the soma and dendrites of the recorded and filled cell, with negligible spillover in the extracellular space. MNs were excluded from analysis if the whole cell patch-clamp recording was stable for <20 min, the cell soma was not clearly intact, or the dendrites were shorter than 20 μm. At the end of each electrophysiology experiment, the slices were fixed in 4% paraformaldehyde (PBS; pH 7.4) overnight, dehydrated through ascending concentrations of methanol and cleared in 1:2 benzyl alcohol (A395, Thermo Fisher Scientific)/benzyl benzoate (0215483990, Millipore). The slices were mounted in depression glass slides, coverslipped and imaged with a Leica TCS SP8 multiphoton confocal microscope equipped with supercontinuum white laser source and single molecule detection hybrid detectors (SMD HyD; Leica). A DFC365FX camera at 2048 × 2048-pixel resolution was used for navigation through the sample. Final images for analysis were taken at 10× from the entire slice, to position the injected neurons and verify its hypoglossal MN by a distinct axon coursing in the intramedullar portion of the XII nerve. Then z-stacks were taken with 20×/0.75 oil-immersion objective, at *z*-step size of 2.4 μm to produce volumes allowing full reconstruction of the biocytin injected MN. In all cases, inclusive reconstruction of the MNs’ dendritic tree required several adjacent volumes to be stitched together. Biocytin injected cells were captured in photon counting regime, an approach allowing for high-dynamic range images to be collected. This was necessary since the injected cell body and the fine distal dendrites represented dramatically different fluorescence signal, which cannot be captured without saturation or missing signal, using more conventional analog photomultipliers. Images were saved in the “Lif” format, then processed and analyzed using Imaris software (Bitplane Inc.).

### Image analysis and morphometric measurements

Several morphologic properties: cell body sizes, regional variation in cell body sizes, dendritic branching, total dendritic length and dendrtic complexity evaluated by Sholl analysis were analyzed after tracing both WT and *LgDel* CNXII protruder and retractor MNs entire dendritic arborization from three-dimensional confocal stacks (Imaris software, Bitplane Inc.). Somatic volume of biocytin filled cell bodies as well as retrograde-labeled CTB-Alexa Fluor 555 and 488 fluorescent cell bodies were directly measured from the confocal-generated volumes. Sholl analysis of branching was performed using an Imaris algorithm for branching analysis. The soma of each labeled cell was identified as the center for this analysis. All dendrites from each cell were traced manually through three dimensions, assisted by the AutoPath protocol. The number of dendritic intersections with a series of concentric spheres at intervals of 10 μm from the soma center point was determine using the Imaris’ Sholl analysis algorithm.

### Data analyses and statistical methods

All electrophysiological data were collected and digitized using Clampex (10.2, Molecular Devices) and analyzed with Clampfit (10.2, Axon Instrument, Molecular Devices, LLC). CNXII rootlet inspiratory bursting activity, MN AP firing and synaptic events were detected using MiniAnalysis (version 6.0.7 Synaptosoft) with the minimal acceptable spike amplitude at 3 V, 60 mV, and 10 pA, respectively. AP firing and synaptic events occurring during inspiratory bursting epoch were defined as bursting related or phasic activity; AP firing and synaptic events not coincident with the bursting period were considered basal activity. Burst-related AP and postsynaptic currents were grouped into 13 consecutive 1-s epochs. The first 3–5 s before burst onset were control (before burst), followed by three 1-s burst epochs and a 3- to 5-s recovery (postburst). All data were within a normal distribution with equal variances using frequency distribution and Bartlett’s corrected statistic test, respectively. Statistical analysis was performed using GraphPad Prism 5.0 (GraphPad Software), Microcal Origin 6.1 (OriginLabs), and Microsoft Excel (Microsoft). Data are presented as mean ± SEM. For WT versus *LgDel* comparisons, we used unpaired Student’s *t* tests. For within group comparisons before, during, and postburst, we used one-way ANOVA with repeated measures and Dunnett’s *post hoc* analysis. Two-way RM ANOVA with Bonferroni *post hoc* tests were applied for between group comparisons. For all comparisons, the significance level was set at *p* < 0.05.

## Results

### Identifying protruder and retractor hypoglossal MNs

Hypoglossal MNs (XII MNs) that innervate protruder and retractor muscles of the tongue are located in the hypoglossal nucleus (CNXII) in the dorsomedial medulla (Fig. 1*A-C*). To differentially examine the electrophysiological properties of protruder and retractor neonatal CNXII in WT and *LgDel*, we identified protruder and retractor CVXII MNs by applying retrograde tracers with different fluorescence characteristics into protruder and retractor muscles at the posterior and lateral aspect of the tongue, respectively (Fig. 1*A*,*B*). Protruder XII MNs were typically located in the ventral CNXII nucleus (Fig. 1*C*, red), whereas retractor XII MNs were typically localized to the dorsal region of the CNXII (Fig. 1*C*, green). The two populations of XII MNs are spatially segregated in CNXII with little overlap (Fig. 1*C* and movie 1). Severing the medial or lateral branch of the hypoglossal nerve abolished labeling of protruder or retractor MNs, respectively (*n* = 3; data not shown). Each recorded XII MN was identified based on its retrograde label as protruder or retractor before electrophysiological recording.

Movie 1. This video shows a detailed 3-D rendering of retrogradely labeled hypoglossal protruder motorneurons (in red) and retractor motorneurons (in green). Protruders were mainly in the rostral (anterior) and ventral parts of the hypoglossal nucleus, whereas retractors were localized more to the posterior (caudal) and dorsal areas within the XII nucleus. 10.1523/ENEURO.0520-19.2020.video.1

### Divergent firing of *LgDel* protruder and retractor MNs

**Figure 2.**
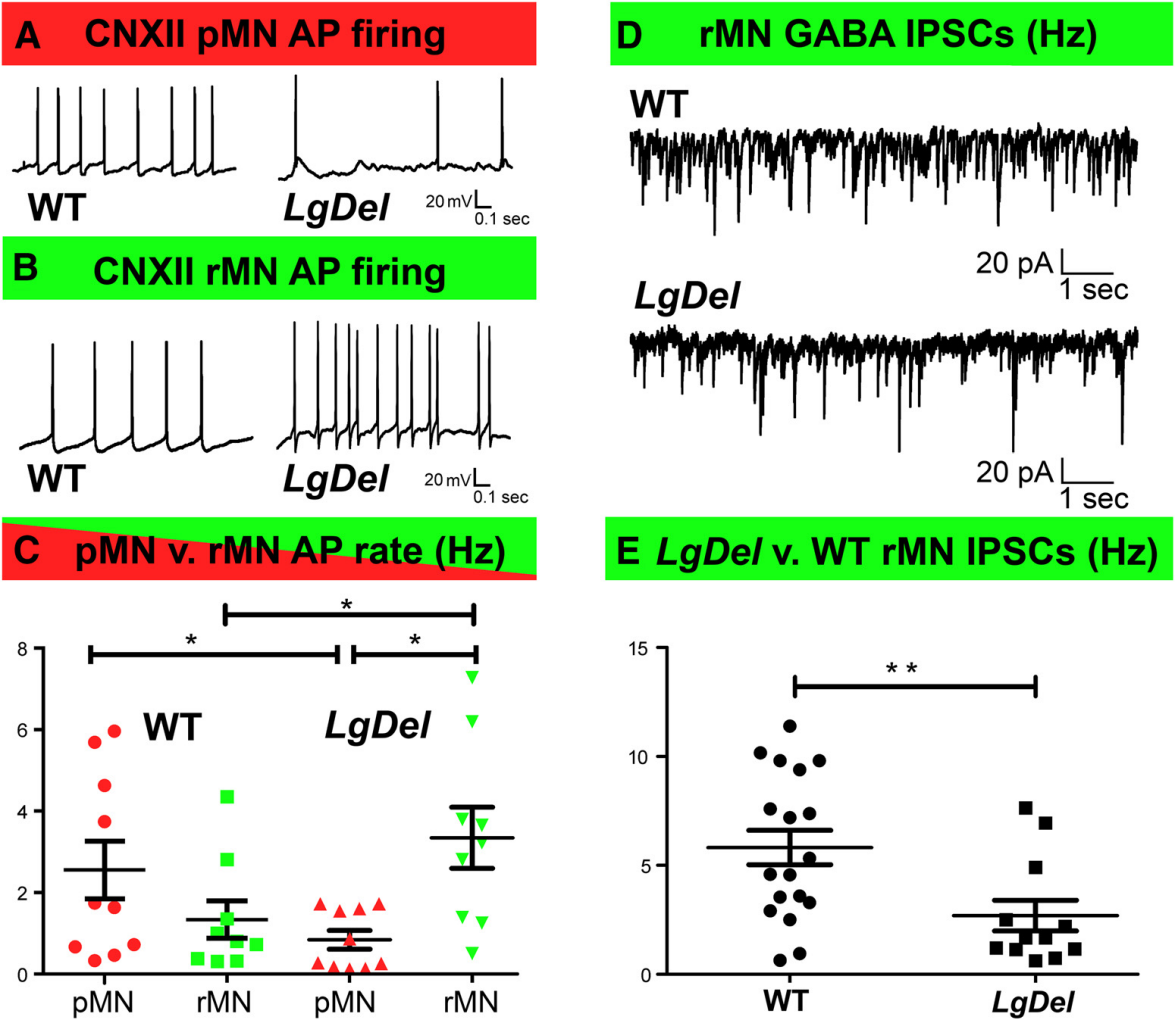
Differences in *LgDel* protruder and retractor MN spontaneous activity. ***A***, Representative traces of spontaneous AP activity of protruder CNXII MNs (pMNs, red) from neonatal WT (left) and *LgDel* (right) pups. ***B***, Spontaneous AP activity of retractor CNXII MNs (rMNs, green) from neonatal WT (left) and *LgDel* (right) pups. ***C***, AP frequency was significantly higher in *LgDel* rMNs compared with both *LgDel* pMNs and WT rMNs as shown in ***C*** (*LgDel* rMN (3.34 ± 0.7 Hz, *n* = 10) compared with *LgDel* pMN (0.84 ± 0.2 Hz, *n* = 9, *p* = 0.011 unpaired Student’s *t* test and WT rMN (1.34 ± 0.4 Hz, *n* = 9, *p* = 0.0398 unpaired Student’s *t* test). The pMN AP firing was statistically higher from WT (2.56 ± 0.7 Hz, *n* = 10) than *LgDel* (0.84 ± 0.2 Hz, *n* = 10, *p* = 0.0434, unpaired Student’s *t* test). ***D***, Typical GABAergic IPSCs events in WT (top) and *LgDel* (bottom) neonatal rMNs. ***E***, Scatter plots show IPSC frequency was significantly greater in WT (5.82 ± 0.8 Hz) versus *LgDel* rMNs (2.70 ± 0.7 Hz, unpaired Students’ *t* test, df = 28, *p* = 0.0099). For all graphs, data expressed as mean ± SEM; statistical comparisons are noted as *p < 0.05, **p < 0.01.

We first compared action potential (AP) firing frequencies for protruder versus retractor CNXII MNs in P3–P6 WT and *LgDel* pups in current clamp mode. In both genotypes, the XII MNs had similar baseline resting membrane potentials, typically between −50 and −65 mV, with AP spike amplitudes of 70–80 mV. The mean spontaneous basal AP frequencies did not differ between WT protruder and retractor XII MNs (Fig. 2*A-C*). In contrast, spontaneous AP frequencies were significantly less in *LgDel* protruder XII MN than in either WT protruder or *LgDel* retractor MNs (Fig. 2*A-C*). Significantly higher firing rates were found in *LgDel* retractor XII MNs than those recorded in either WT retractors or *LgDel* protruders (Fig. 2*A-C*). Thus, heterozygous 22q11.2 gene deletion is associated with significant alterations in basal firing frequencies of CNXII MNs innervating protruder and retractor tongue muscles.

To further identify possible causes for differential *LgDel* CNXII retractor MN AP firing frequency, we studied excitatory and inhibitory synaptic inputs to CNXII MNs. There were no significant differences in either frequency nor amplitude of EPSCs that could possibly contribute to increased firing rates *observed in LgDel* CNXII retractor MN (data not shown). In contrast, frequency, but not amplitude, of GABAergic IPSCs was significantly diminished in *LgDel* MN compared with WT (Fig. 2*D*,*E*). This latter data suggests that, reduced GABAergic inhibition might be at least in part a contributing factor for the enhanced *LgDel* retractor MN firing.

### Phasic CNXII MN activity during inspiratory-related bursts

**Figure 3.**
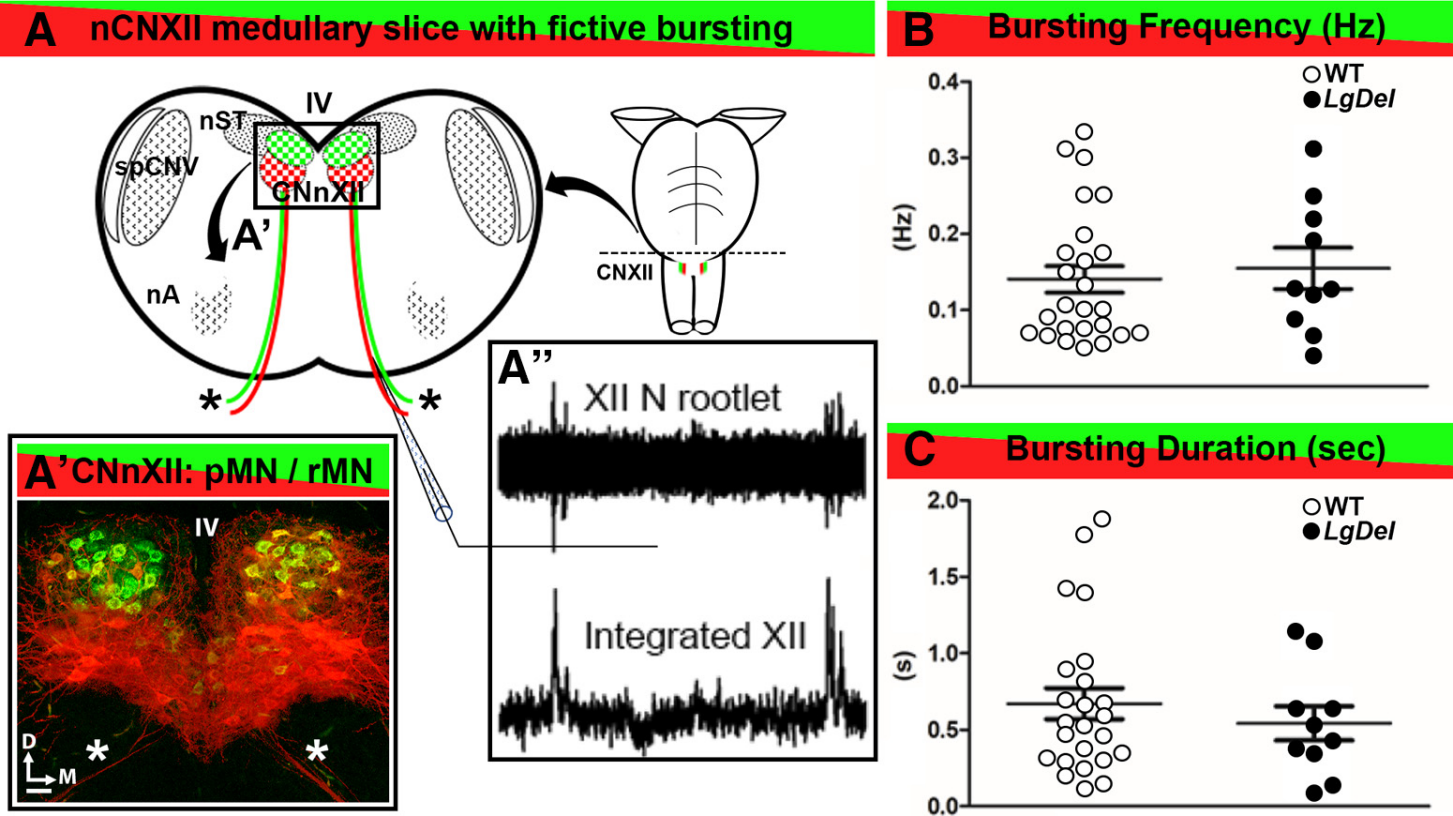
Spontaneous fictive respiratory bursts in WT and *LgDel* CNXII slices. ***A***, A schematic of a lower medulla section taken at the level of CNXII showing the nucleus (CNXII, box) ventral to the fourth ventricle (IV), segregated populations of pMNs (red) and rMNs (green), CNXII axons extending ventrally exit as CNXII rootlets (asterisks), and adjacent cranial nuclei including the nucleus of the solitary tract (nST), the spinal trigeminal nucleus (spCNV), and nucleus ambiguous (nA). ***A****’*, A confocal image of CNXII showing the segregation of the pMNs (red), rMNs (green), and CNXII axons extending ventrally (asterisks). Scale bar: 50 μm. D: dorsal, M: medial. ***A’’***, Spontaneous inspiratory bursting activity recorded from hypoglossal nerve rootlets using a suction electrode (top), and represented as an integrated electrophysiological signal (right). ***B***, Scatterplot of spontaneous inspiratory related bursting frequency recorded from WT and *LgDel* slices. ***C***, Scatter plot of burst durations from WT and *LgDel* slices. Burst frequency and duration were not significantly different in the two genotypes; WT (*n* = 25) and *LgDel* (*n* = 10) animals, *p* > 0.05, unpaired two-tailed Student’s *t* test.

Tongue movements are coordinated with respiratory phases to optimize the suck/breathe/swallow cycle by ensuring that a physiological separation of the air and food traffic (Matsuo and Palmer, 2015; Maynard et al., 2020). Accordingly, we assessed the putative rhythmic respiratory activity which can be confined within the thick neonatal slices (Wang et al., 2005) on protruder and retractor XII MN firing (Fig. 3*A*,*A’’*). This slice retains a characteristic rhythmic inspiratory-related motor discharge in CNXII MN (Fig. 3*A*,*A’*). Spontaneous inspiratory-related group activity of CNXII was recorded by a suction electrode from hypoglossal nerve rootlets (Fig. 3*A’’*). Hypoglossal rootlet activity was amplified 50,000 times, filtered (10- to 300-Hz bandpass), and integrated electronically (τ = 50 ms; Fig. 3*A’’*). No significant differences were detected when comparing mean inspiratory burst frequency between WT and *LgDel* (*p* = 0.6664; two tail unpaired Student’s *t* test; Fig. 3*B*) or burst duration (*p* = 0.2015, two tail unpaired Student’s *t* test; Fig. 3*C*). In all subsequent experiments, no procedures or manipulations were done or applied to increase the incidence/magnitude of respiratory modulation (such as pH manipulations).

**Figure 4.**
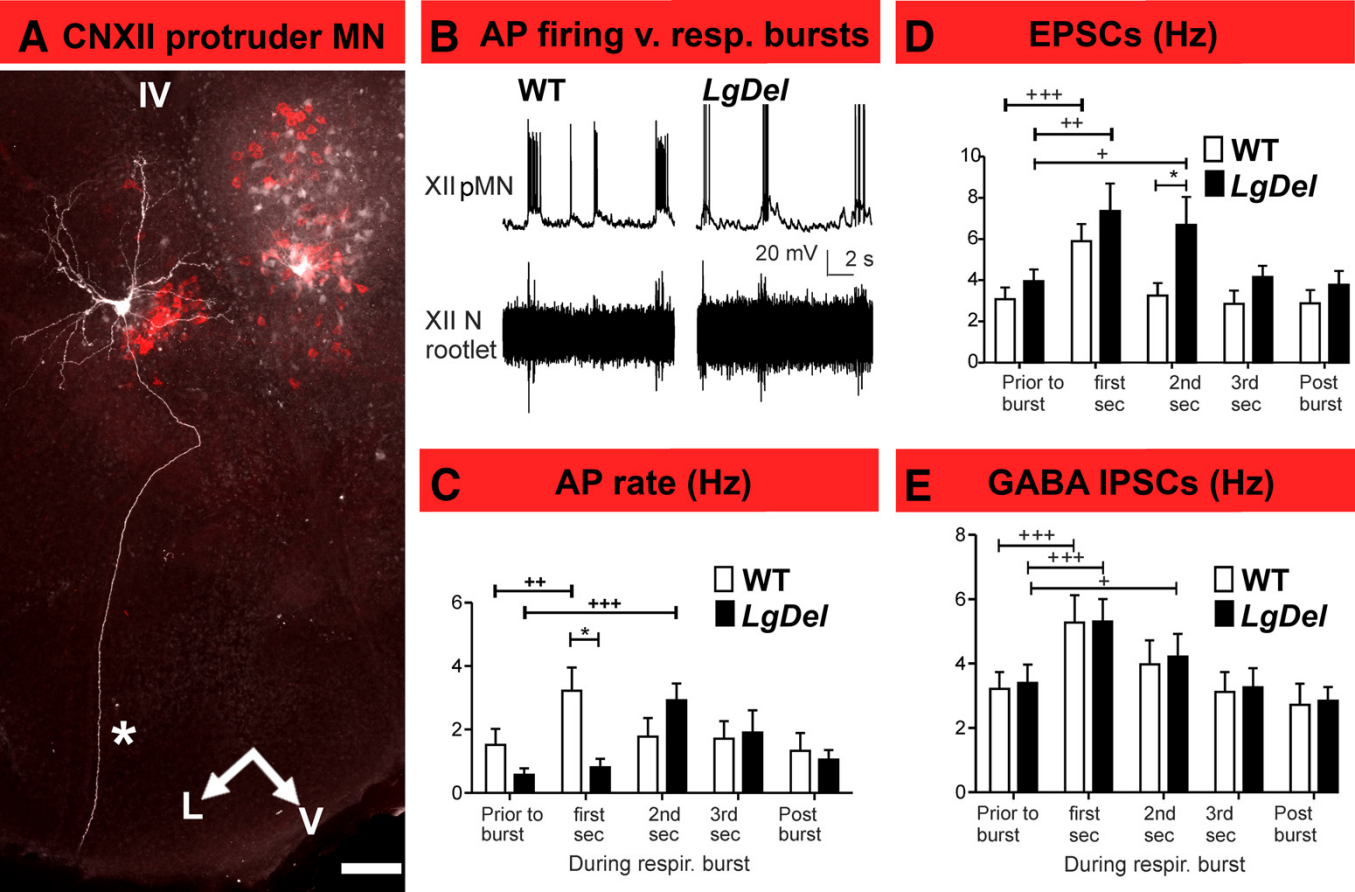
Delayed inspiratory burst-related *LgDel* protruder MN activity. ***A***, A confocal image of a pMN labeled by intracellular injection with biocytin, after identification using retrograde tracing with CTB-Alexa Fluor 555 (red). The asterisk indicates the single axon extending from this pMN to exit in the ventromedial region of the posterior medulla. Scale bar: 100 μm. V: ventral, L: lateral, IV: fourth ventricle. ***B***, Representative traces showing the relationship between pMN AP activity (top traces) and fictive respiratory bursts recorded at CNXII rootlets (bottom traces) in WT versus *LgDel* neonatal mice. ***C***, Histograms of AP firing in WT (white bars) and *LgDel* (black bars) pMNs before, during, and after inspiratory bursts. WT protruder MN AP firing increased in the first 1-s postburst interval (prior: 1.52 ± 0.5 Hz, first second 3.22 ± 0.7 Hz, one-way repeated measures ANOVA with Dunnett’s multiple comparison *post hoc* test, *p* = 0.0014, DFn = 4, DFd = 49, *F* = 5.538), whereas *LgDel* protruder MNs AP firing increased in the second, but not first, 1-s postburst interval (prior: 0.7 ± 0.1 Hz, first second 0.8 ± 0.1 Hz, second second 2.9 ± 0.7 Hz, one-way repeated measures ANOVA with Dunnett’s multiple comparison *post hoc* test, *p* = 0.0009, DFn = 4, DFd = 39, *F* = 6.408). The AP firing was higher in WT (3.22 ± 0.7 Hz) than in *LgDel* (0.8 ± 0.1 Hz) at the first 1-s postburst interval (two-way ANOVA with Bonferroni *post hoc* test, *p* < 0.05). ***D***, Histograms of EPSC frequency in WT and *LgDel* pMNs before, during, and after inspiratory bursts. EPSC frequency from WT and *LgDel* pups increases significantly during the first 1-s postinspiratory burst interval (WT: prior: 3.1 ± 0.5 Hz, first second 5.9 ± 0.8 Hz, second second 3.3 ± 0.5 Hz, one-way repeated measures ANOVA with Dunnett’s multiple comparison *post hoc* test, DFn = 4, DFd = 59, *F* = 15.76, *p* < 0.0001; *LgDel* protruder MNs: prior: 3.97 ± 0.5 Hz, first second 7.37 ± 1.3 Hz, second second 6.7 ± 1.3 Hz, one-way repeated measures ANOVA with Dunnett’s multiple comparison *post hoc* test, DFn = 4, DFd = 44, *F* = 6.909, *p* = 0.0004); however, *LgDel* pMN EPSC frequency continued at significantly elevated levels through the second 1-s postburst interval compared with WT (6.7 ± 1.3 Hz in *LgDel* vs 3.3 ± 0.5 Hz in WT, *p* < 0.05, two-way ANOVA with Bonferroni *post hoc* test). ***E***, GABAergic IPSC frequency histograms of WT and *LgDel* protruder MNs. IPSC frequency increased significantly during the first 1-s postburst interval in WT (prior: 3.2 ± 0.5 Hz, first second 5.3 ± 0.8 Hz, second second 4.0 ± 0.7 Hz, one-way repeated measures ANOVA with Dunnett’s multiple comparison *post hoc* test, DFn = 4, DFd = 44, *F* = 13.55, *p* < 0.0001) and *LgDel* pups (prior: 3.7 ± 0.5 Hz, first second 5.8 ± 0.6 Hz, second second 4.7 ± 0.6 Hz, one-way repeated measures ANOVA with Dunnett’s multiple comparison *post hoc* test, DFn = 4, DFd = 49, *F* = 16.96, *p* < 0.0001). Data expressed as mean ± SEM; Comparison between WT and *LgDel* group; *p < 0.05. Comparison among different time points within same group; +*p* < 0.05, ++*p* < 0.01, +++*p* < 0.001.

Firing rate significantly increased in both WT and *LgDel* protruder XII MNs (Fig. 4*A*) during inspiratory bursts (Fig. 4*B*) compared with the baseline firing rate. Nevertheless, temporal burst dynamics differ. WT protruder AP firing rates increased significantly only within the first 1 s after bursts’ onset (Fig. 4*C*), whereas in *LgDel*, protruder MN the firing did not increase significantly during the first 1 s of the burst but did significantly increase in the second epoch (Fig. 4*C*). To identify possible underlying mechanisms for delayed *LgDel* protruder MN excitation, we examined inspiratory-related EPSCs and IPSCs. WT and *LgDel* XII MN demonstrated similar and significant increase in EPSC frequency during the first 1 s following the onset of the inspiratory burst (Fig. 4*D*). In WT protruders, EPSC frequency declined to baseline by the second epoch, whereas in *LgDel* XII MNs, the EPSC frequency remained significantly elevated (Fig. 4*D*). IPSC frequency also increased significantly during the first 1 s after the burst onset in both WT and *LgDel* pups (Fig. 4*E*). IPSC frequency only increased in the 1-s postburst epoch in protruder XII MNs from WT animals, whereas in *LgDel* protruder XII MNs IPSC frequency was increased in the 1-s postburst epoch and remained significantly elevated extending to the second epoch (Fig. 4*E*).

**Figure 5.**
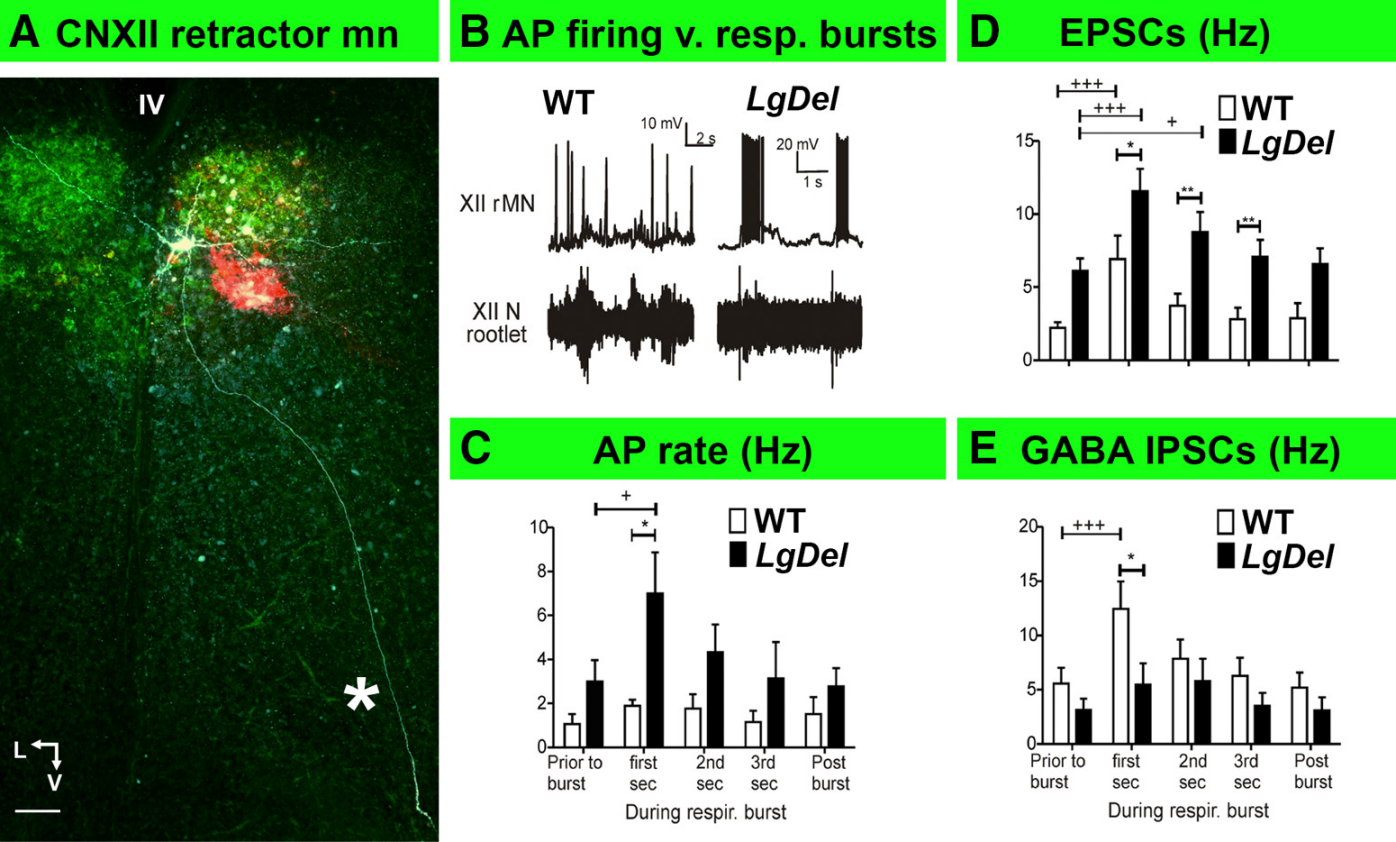
Increased inspiratory burst-related activity in *LgDel* retractor CNXII MNs. ***A***, A confocal image of a rMN labeled by intracellular injection with a biocytin (fluorophore wavelength), after identification using retrograde tracing with CTB-Alexa Fluor 488 (green). The asterisk indicates the single axon extending from this rMN to exit in the ventromedial region of the posterior medulla. Scale bar: 50 μm. V: ventral, L: lateral, IV: fourth ventricle. ***B***, Representative traces of rMN AP activity (top traces) from WT and *LgDel* mouse pups in response to fictive inspiratory bursts (bottom traces). ***C***, Histograms of rMN AP frequency in WT (white bars) and *LgDel* (black bars). rMN AP frequency differs through five distinct epochs before, during, and after inspiratory bursts. In *LgDel* rMNs AP activity increased from 3.0 ± 0.9 Hz before bursts to 7.0 ± 1.8 Hz in the first 1-s interval following burst initiation (one-way repeated measures ANOVA with Dunnett’s multiple comparison *post hoc* test, DFn = 4, DFd = 34, *F* = 3.17, *p* = 0.0317). This increase was significantly greater than the increased AP frequency in WT rMNs: (WT 1.8 ± 0.2 Hz; *LgDel* 7.0 ± 1.8 Hz, two-tailed unpaired Student’s *t* test *p* = 0.036. DFn = 6, DFd = 7). ***D***, Histograms of EPSC frequency in WT and *LgDel* pMNs before, during, and after inspiratory bursts. *LgDel* retractor EPSC frequency increased significantly during the first (11.6 ± 1.5 Hz) and second 1-s postburst intervals (8.8 ± 1.3 Hz) compared with levels before the burst (6.1 ± 0.8 Hz, one-way RM ANOVA DFn = 4, DFd = 44, *F* = 8.392, *p* < 0.0001, with Dunnett’s multiple comparison test). *LgDel* rMN EPSC frequency was greater than that in WT for each of the three 1-s postburst intervals (two-way ANOVA, DFn = 4, DFd = 100, *F* = 6.3 with Bonferroni multiple comparisons: first second 6.9 ± 1.2 Hz in WT, 11.6 ± 1.5 Hz in *LgDel*; *p* = 0.04; second second 3.7 ± 0.8 Hz in WT, 8.8 ± 1.3 Hz in *LgDel*; *p* = 0.006; third second 2.8 ± 0.7 Hz, WT, 7.1 ± 1.1 Hz, *LgDel*; *p* = 0.007). ***E***, Histograms of GABAergic IPSC frequency in WT and *LgDel* pMNs before, during, and after inspiratory bursts. No significant change in GABAergic IPSC frequency in *LgDel* rMNs over the entire duration of the burst (DFn = 4, DFd = 39, *F* = 2.328, *p* = 0.0807, one-way ANOVA). WT rMNs GABAergic IPSC frequency was increased significantly from 5.6 ± 1.0 Hz (before the burst) to 12.4 ± 2.5 Hz (during the first 1-s postburst interval, one-way RM ANOVA with Dunnett’s multiple comparison test, DFn = 4, DFd = 39, *F* = 8.596, *p* = 0.0001). The rMN IPSC frequency was greater in WT than *LgDEl* (12.4 ± 2.5 Hz in WT, 5.5 ± 1.9 Hz, two-way ANOVA with Bonferroni multiple comparisons, *p* = 0.0270, *F* = 2.92, DFn = 4, DFd = 70). Data expressed as mean ± s.e.m; Comparison between WT and LgDel group *p < 0.05 and **p < 0.01. Comparison among different time points within same group +*p* < 0.05, +++*p* < 0.001.

AP frequency did not change significantly in WT retractor XII MN (Fig. 5*A*) during putative inspiratory bursts (Fig. 5*B*,*C*). EPSC frequency increased during inspiratory bursts in WT retractor XII MNs (Fig. 5*D*); however, this was apparently balanced by increased GABAergic IPSC frequency, resulting in no change in firing of WT retractors (Fig. 5*C–E*). In contrast, *LgDel* retractor MN activity increased with putative inspiratory bursts. This was accompanied by a significantly greater increase in EPSC frequency (compared with WT) with no inhibitory counterbalance during each of the 3-s burst epochs (Fig. 5*D*). GABAergic IPSC frequency did not change in *LgDel* retractors during the burst (Fig. 5*E*). Divergent integration of inspiratory burst and retractor firing activity in *LgDel* pups likely reflects disrupted excitation/inhibition synaptic inputs to *LgDel* retractor CNXII MNs.

### Divergent size and structure of *LgDel* retractor XII MNs

The electrophysiological differences between WT and *LgDel* retractor and protruder XII MNs may result in part from morphologic differences between these neurons. Somatic size for example determines the integrative properties, while the complexity of the dendritic tree is likely to determine the synaptic quantity and quality of synaptic inputs. Overall, the neuronal morphology could impact integration of presynaptic inputs, postsynaptic responses, and circuit properties. Thus, we analyzed cell body volumes and dendritic morphology of WT and *LgDel* retractor and protruder XII MNs labeled by intracellular injection of biocytin, using slices that also pre-labeled with retrograde tracers for protrude and retractor identification.

**Figure 6.**
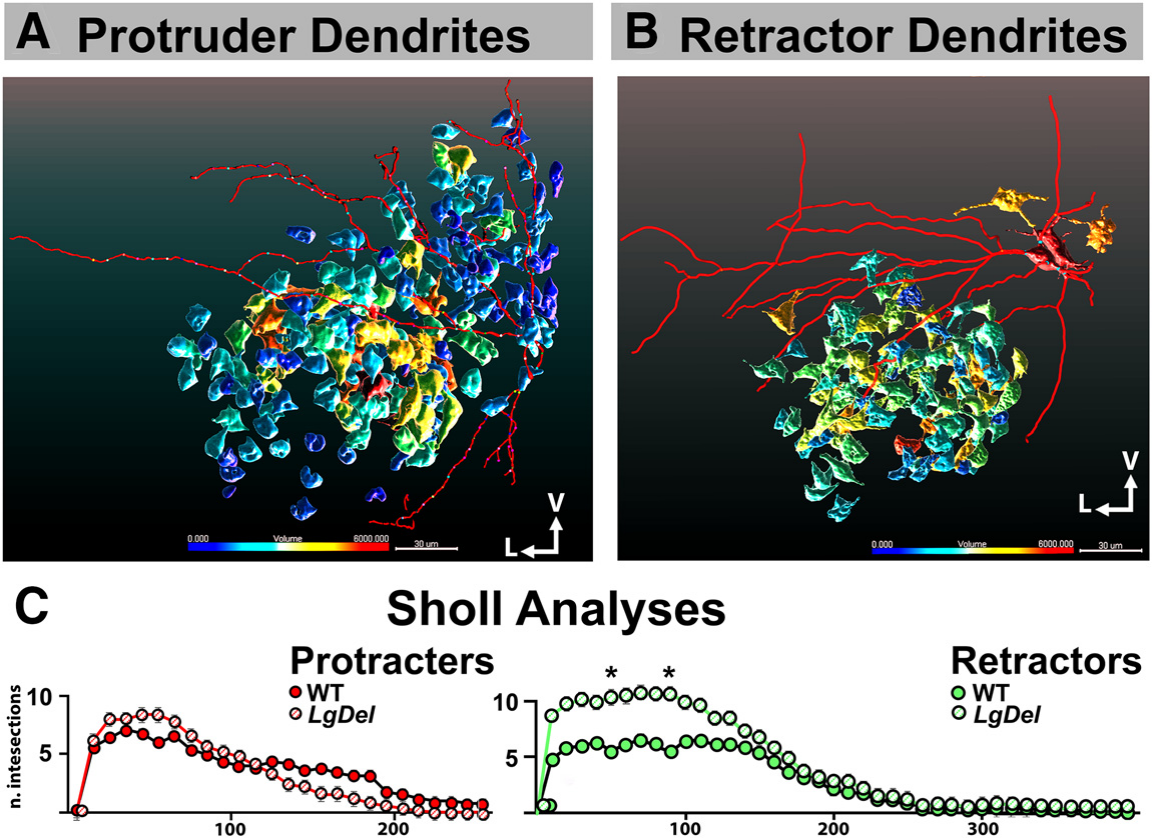
Changes in retractor CNXII MNs cell soma and dendritic arborization in the hypoglossal nucleus of *LgDel* mouse pups. ***A***, ***B***, Representative confocal image renderings of a CNXII pMN (left) and rMN (right) after patch clamp recording followed by intracellular-labeled cell dendritic structure and soma volume in WT (pMN, left) or *LgDel* (rMN, right). The color coding illustrating the size of cell body volumes; V, ventral, L, lateral. Scale bar: 30 μm. ***C***, Scholl analyses of CNXII pMN (left) and rMN (right) dendritic branching showing statistic difference only in rMN but not in pMN, the total numbers of dendritic intersections at 50- and 90-μm radii were significantly greater in *LgDel* than WT retractor MNs; at 50 μm: 5.57 ± 0.4 in WT, 10.29 ± 1.4 in *LgDel, t* = 3.339, *p* < 0.05; at 90 μm: WT 5.42 ± 1.9, *LgDel* 10.58 ± 2.1, *t* = 3.642, *p* < 0.05, two-way RM ANOVA with Bonferroni *post hoc* tests. Data presented as Mean ± SED; *p < 0.05.

We first assessed the cell body volume distribution by first applying 3-D segmentation of the retrogradely labeled XII protruder and retractor WT and *LgDel* MN labeled by intracellular biocytin in WT and *LgDel* throughout the nucleus. There were no significant differences in volume of either XII MN class in WT and *LgDel* (Fig. 6*A*,*B*). We next assessed the extent, orientation, branching properties of WT versus *LgDel* XII MN dendrites. The predominant orientation of XII MN dendrites in both genotypes was in the transverse plane. Some XII MN dendrites extended into the adjacent intercalated nucleus of Staderini, ventral part of the medullary reticular nucleus, or the gigantocellular reticular nucleus. However, there was relatively less rostro-caudal dendritic extension (Fig. 6*A*,*B*). Sholl analysis, reflecting dendritic complexity and the maximum distance from the soma, of WT (*n* = 5) and *LgDel* (*n* = 6) protruder XII MNs (Fig. 6*C*) did not reveal significant difference among the two genotypes (190 ± 30 μm for WT and 178 ± 16 μm for *LgDel*). The total dendritic length did not differ for WT (1479 ± 235 μm, *n* = 5) versus *LgDel* (1105 ± 187 μm, *n* = 8) protruder CNXII MNs.

In contrast, the dendritic architecture of retractor CNXII MNs differed between the WT and *LgDel*. The total numbers of dendritic intersections at 50- and 90-μm radii from the cell soma were significantly greater for *LgDel* versus WT retractor MNs (two-way ANOVA with Bonferroni *post hoc* correction, *n* = 7 WT, *n* = 7 *LgDel*, *F* = 8.942; at 50 μm: 5.57 ± 0.4 WT vs 10.29 ± 1.4 *LgDel*, *p* < 0.05; at 90 μm: 5.42 ± 1.9 for WT vs 10.58 ± 2.1 *LgDel; p* < 0.05; Fig. 6*C*). The maximal distance from the soma for retractor dendritic intersections, however, did not differ significantly for *LgDel* when compared with WT retractor XII MNs (199 ± 38 μm WT vs 260 ± 15 μm *LgDel*, unpaired two tailed Student’s *t* test, *p* = 0.1543, *n* = 7 in both groups).

## Discussion

The coordination of XII protruder and retractor MN activity is altered in *LgDel* pups, in which several aspects of S/F/S, including nasopharyngeal aspiration are parallel to pediatric dysphagia seen with high frequency in infants and toddlers with 22q11.2DS. The basal spontaneous and inspiration-related AP firing rates as well as synaptic properties of protruder versus retractor XII MNs in *LgDel* mouse pups differ from those in WT. The physiological differences in *LgDel* retractor MNs are more substantial than those between other XII MN classes. In addition, *LgDel* retractor MN dendritic arbors are more branched. These distinctions in physiological, network, and cellular properties of *LgDel* CNXII retractor and protruder MNs may underlie anomalous coordination of tongue movement critical for S/F/S early in life, as well as formation and oropharyngeal transit of food boluses subsequently. Thus, pediatric dysphagic symptoms that result from heterozygous 22q11.2 gene deletion may reflect altered development and function XII MNs and circuits in addition to anomalies in differentiation of oropharyngeal structures critical for optimal S/F/S at birth and throughout life.

We found that the activity of retractor hypoglossal MNs in *LgDel* mouse pups is abnormally elevated compared either to *LgDel* protruder MNs or WT retractor MNs. This is consistent with elevated firing frequency reported in *LgDel* CNXII MNs whose projection targets were not identified (Wang et al., 2017). In addition to augmented firing, dendritic branching increases in *LgDel* retractor MNs suggesting that increased morphologic complexity could be a substrate for enhanced excitatory synaptic inputs observed in these neurons.

WT retractor XII MNs excitatory synaptic inputs during putative inspiratory bursts are counterbalanced by enhanced inhibitory inputs, which presumably act to maintain consistent firing frequencies during inspiratory bursts. In contrast, *LgDel* retractor XII MNs firing increases significantly during inspiratory-related bursts. This divergent output to retractor tongue muscles is likely mediated by both the abnormally prolonged increase in EPSC frequency (lasting 3 s in *LgDel*, but only 1 s in WT), and absence of an opposing increase in inhibitory GABAergic inputs. The resulting protracted period of higher frequency firing because of this change in excitatory/inhibitory (E/I) balance may increase retraction of the tongue during the suck/breathe/swallow cycle, reduce milk flow into the esophagus, diminish total milk intake during infancy and disrupt food bolus formation at later ages. These changes could also prevent appropriate tongue position during S/F/S, blocking the epiglottis from closing to protect the nasopharynx from aspiration (Maynard et al., 2020) and may be important in dysregulated tongue coordination and S/F/S difficulties in *LgDel* mouse pups (Karpinski et al., 2014).

This selective disruption of E/I balance may be a direct, local effect of 22q11.2 deletion on the differentiation of XII MNs and associated interneurons (Karpinski et al., 2014) or an indirect consequences of altered target peripheral oropharyngeal differentiation associated with 22q11.2 deletion (Scambler, 2010). Substantial inhibitory and excitatory afferent inputs, as well as reticular pre-MNs to the hypoglossal nucleus (Holstege et al., 1977; Borke et al., 1983; Travers and Norgren, 1983; Dobbins and Feldman, 1995) had been reported. The local circuit interneurons within the hypoglossal nucleus act in concert with other afferents terminating on hypoglossal motoneurons and likely on the interneurons as well had been found in rat (Li et al., 1993; Popratiloff et al., 2001; Williams et al., 2019) and mouse (Ireland et al., 2004; Oku et al., 1999). Hypoglossal MN dendritic arbors continue to grow and differentiate after birth (Kanjhan et al., 2016), and are sensitive to altered GABAergic signaling (Fogarty et al., 2013, 2017). Our morphologic analysis of *LgDel* versus WT protruder and retractor MNs suggests that some changes may reflect altered interactions between XII MNs and dysmorphic targets. Indeed, substantial changes in *LgDel* retractor MN synaptic and circuit properties are accompanied by substantial changes in dendritic size and branching. Further assessment of 22q11.2 gene dosage-dependent modulation of CNXII MN and interneuron differentiation, tongue muscle differentiation, and resulting afferent/target interactions that might influence circuit differentiation will further define pathogenic vulnerability of brainstem circuits underlying perinatal dysphagia in 22q11.2DS.

*LgDel* protruder XII MNs also are significantly different their WT counterparts. Spontaneous AP firing frequency is significantly less in *LgDel* protruder XII MNs than that in WT. During putative inspiratory related bursting in WT, protruder AP firing only increases during the first second from the start of the burst, whereas in *LgDel*, protruder firing activity is delayed. *LgDel* protruder AP firing increases significantly in the second 1-s epoch after the start of the burst, likely because of delayed excitatory inputs to *LgDel* protruders. The combined consequences of significantly enhanced, temporally anomalous CNXII retractor MN firing and temporally dysregulated CNXII protruder MN activity may degrade the coherence of coordinated neural control of tongue movement critical for optimal S/F/S.

We did not examine the function of protruder and retractor muscles in WT or *LgDel* mouse pups *in vivo*; however, additional observations (Welby et al., 2020) indicate that tongue movement and related behaviors are altered in older *LgDel* mice. Disrupted coordination of the suck/swallow/breath sequence in infants and toddlers with 22q11.2DS slows or interrupts feedings because of gagging and/or regurgitation (Eicher et al., 2000). Older children with 22q11.2DS who can eat solid food often refuse to eat and/or gag, indicating aversive or impaired oral transport (Eicher et al., 2000; Rommel et al., 2008). Dysarthria, slurred or slow speech, is also prevalent in these children and thought to reflect tongue or throat muscle dysfunction (Persson et al., 2017; Baylis and Shriberg, 2019). Obstructive sleep apnea, where the tongue closes the upper airway repetitively during sleep, is more common in 22q11.2DS (Kennedy et al., 2014). Differential dysregulation of *LgDel* protruder versus retractor hypoglossal MN outputs is a prime candidate for disrupted S/F/S as well as additional oromotor dysfunction. Although altered protruder MN activity likely complicates S/F/S in 22q11.2DS, retractor MN output changes are more pronounced. These results might predict that E/I imbalance and elevated retractor hypoglossal MN firing alters tongue compression and backward movement (Fregosi and Ludlow, 2014), thus threatening both food intake efficiency and airway safety in infants and toddlers with 22q11.2DS. While it would be premature to attempt to target new therapies for feeding, swallowing and vocalization difficulties in 22Q11.2DS patients based on these results, our work indicates that targeted inhibition of retractor hypoglossal MNs or muscles could provide a foundation for improved perinatal oromotor function.

## Synthesis

Reviewing Editor: Robert Kalb, Northwestern University Decisions are customarily a result of the Reviewing Editor and the peer reviewers coming together and discussing their recommendations until a consensus is reached. When revisions are invited, a fact-based synthesis statement explaining their decision and outlining what is needed to prepare a revision will be listed below. The following reviewer(s) agreed to reveal their identity: R. Alberto Travagli.

Strong work well revised
